# Supplementary material for: Content-rich biological network constructed by mining PubMed abstracts
Source: BMC Bioinformatics. 2004 Oct 8;5:147. doi: 10.1186/1471-2105-5-147 (PMC528731; doi:10.1186/1471-2105-5-147)
Supplement: Additional File 2 — The original results of the above study (non-essential files are deleted to keep the file size under the limit set by BMC bioinformatics). [file 1471-2105-5-147-S2.bz2 › chilibotAdditionalFile2/dip05/731ID11983079E18089/html/HSCA_NIFU.html]

 


 **HSCA** and **NIFU** 
  
Found 3 abstracts in PubMed, retrieved 3.  
 

 What does Google say? 
 PDF only 
| .edu only 

---

**Interactive relationship** (e.g. stimulation, inhibition, etc)

**Stimulatory relationship**- A charge mutant containing the motif sequence of  **NifU** , IscU V102E, did not bind the ATP or ADP states of Hsc66  [ **HSCA** ]  but did bind Hsc20 and weakly stimulated Hsc66  [ **HSCA** ]  ATPase in the presence of the co chaperone.  Ref: 12871959 J Biol ChemJ Biol Chem,
- IscU, a  **NifU**  like  [ **NIFU** ]  Fe S escort protein, binds to and stimulates the ATPase activity of Hsc66  [ **HSCA** ] , a hsp70 type molecular chaperone.  Ref: 11053447 J Biol Chem, 2001
**Neutral relationship**
